# Functional organic fertilizers can alleviate tobacco (*Nicotiana tabacum* L.) continuous cropping obstacle *via* ameliorating soil physicochemical properties and bacterial community structure

**DOI:** 10.3389/fbioe.2022.1023693

**Published:** 2022-10-20

**Authors:** Dan Chen, Mei Wang, Gang Wang, Yujie Zhou, Xiaoe Yang, Jiangzhou Li, Cuiping Zhang, Kuai Dai

**Affiliations:** ^1^ Ministry of Education (MOE) Key Laboratory of Environment Remediation and Ecological Health, College of Environmental and Resource Sciences, Zhejiang University, Hangzhou, China; ^2^ Yuxi Tobacco Company, Ltd. of Yunnan Province, Yuxi, China

**Keywords:** functional organic fertilizers, tobacco continuous cropping obstacle, soil physicochemical property, soil bacterial community, sustainable agriculture

## Abstract

Continuous cropping obstacle (CCO) in tobacco is a prevalent and intractable issue and has not yet been effectively solved. Many researchers have favored exploring environmentally friendly and sustainable solutions to CCO (e.g, the application of (bio-) organic fertilizers). Therefore, to study the effects of functional organic fertilizers (FOFs) on tobacco CCO, we applied five types of fertilizers in a tobacco continuous cropping field with red soil (i.e., CF: tobacco-special chemical fertilizers; VOF: vermicompost-based FOF; HOF: humic acid-based FOF; WOF: wood biochar-based FOF; COF: compound FOF). The tobacco plant agronomic traits, leaf yield, economic value, and chemical quality (nicotine, total sugar, K_2_O, Cl contents, etc.) were evaluated *via* the continuous flow method. Meanwhile, we determined rhizosphere soil physicochemical properties, phenolic acids content, and bacterial community diversity by high-throughput sequencing. The results show that FOFs improved the tobacco plant agronomic traits, leaf yield (by 2.9–42.8%), value (by 1.2–47.4%), and chemical quality when compared with CF. More content of NH_4_^+^-N, available P, and available K were discovered in the rhizosphere soil in VOF, HOF, and WOF. The rhizosphere sinapic acid and total phenolic acids content declined in the FOF treatments (1.23–1.56 and 7.95–8.43 mg kg^−1^ dry soil, respectively) *versus* those in the CF treatment (2.01 and 10.10 mg kg^−1^ dry soil, respectively). Moreover, the rhizosphere bacterial community structure changed under FOF functions: the beneficial microbes *Actinobacteria, Firmicutes*, *Streptomyces,* and *Bacillus* increased, and the harmful microbes *Acidobacteria* and *Gemmatimonadota* decreased in abundance. There was a positive correlation between the tobacco leaf yield and soil NH_4_^+^-N, TC content, and the relative abundance of *Proteobacteria* and *Actinobacteriota*. In summary, the application of VOF and WOF is a modest, practical, and environmentally friendly strategy to alleviate tobacco CCO from the standpoint of recycling solid waste.

## 1 Introduction

Due to the limitation of arable land, the drive for high benefits, and the lack of rational planting, crop continuous cropping (CC) in the same field is a serious and widespread phenomenon in China, and even throughout the world (Zhang et al., 2013; Yuan et al., 2014). Crop CC is often accompanied by an increase in seedling lesions, death, soil-borne diseases, and a decrease in crop yield and quality. This phenomenon is also called continuous cropping obstacles (CCOs) (Zhang et al., 2013). It is reported that over 20% of agricultural land has been threatened by the negative consequences of CC, which has caused enormous economic losses and hindered sustainable agricultural development (Bonner and Galston, 1944; Bai et al., 2019). The crops prone to CCO include pepper (*Capsicum annuum* L.) (Mao and Jiang, 2021), tomato (*Lycopersicon esculentum* Miller) (Zhao et al., 2020), tobacco (*Nicotiana tabacum* L.) (Chen et al., 2018a; Bai et al., 2019), American ginseng (*Panax quinquefolius* L.) (Liu et al., 2020), peanut (*Arachis hypogaea* L.) (Li et al., 2019), cucumber (*Cucumis sativus* L.) (Zhou and Wu, 2012), apple (*Malus pumila* Mill.) (Yin et al., 2016), and so on. Tobacco is one of the most well-characterized economic crops sensitive to CC. Approximately one-third of the world’s tobacco is planted in China, and China is the biggest country producing and consuming tobacco (Zou et al., 2018). Planting tobacco has been the primary income for millions of farmers in China, particularly in poor areas such as Yunnan and Guizhou provinces. However, CCO in tobacco production is also prevalent, which has caused huge economic losses, constrained the implementation of intensive production, and has been listed as one of the important issues and challenges to be addressed in the tobacco industry (Niu et al., 2017; Chen et al., 2018b).

There are three main well-known causes for crop CCO: deterioration of the soil’s physicochemical properties, an accumulation of crop allelopathic substances (primarily phenolic acids-PA), and a change of soil microbial community structure, which can be collectively referred to as an imbalance of soil micro-ecology environment (Zhou and Wu, 2012; Yin et al., 2016; Chen W et al., 2018; Bai et al., 2019). Based on this, adjusting the soil’s unbalanced micro-ecology environment to its former healthy condition is a basic and crucial concept to alleviate crop CCO. Tobacco needs to absorb large quantities of nutrient elements during the growth period, especially for N, P, and K. Tobacco farmers usually apply tobacco-special compound chemical fertilizers. There is no doubt that chemical fertilizers have the advantage of being relatively cheap and quick acting, and the application of chemical fertilizers in agriculture greatly increases crop productivity and alleviates the international food crisis. However, several environmental problems have occurred alongside the over-application of chemical fertilizers, such as soil acidification, soil hardness, nutrient imbalance, excessive greenhouse gas emissions, and eutrophication of lakes and rivers after N and P losses by runoff, which threatened the sustainable utilization of soil and mineral resources (Diaz and Rosenberg, 2008; Guo et al., 2010; Jiao et al., 2016; Zhang et al., 2016). Therefore, a series of more sustainable and environmentally friendly fertilizers are urgently required to replace chemical fertilizers in a rational way. Organic fertilizers can replace chemical fertilizers, improve soil fertility, increase enzyme activity, adjust soil microbial community structure, and reduce the occurrence of soil-borne diseases, thus alleviating crop CCO and increasing crop yield and quality (Lv et al., 2011; Lazcano et al., 2013; Liu et al., 2018; Li et al., 2019). Based upon these excellent features, organic fertilizers offer a broad prospect for application.

Rapeseed cakes and mushroom residues are two by-products of agricultural activities that are available in large quantities and need to be properly utilized. Due to their high N/P/K contents and other micro-elements, they can be the main raw resources of organic fertilizers. Vermicompost is an organic compost product that is derived from organic matter decomposition and degradation processes by the action of earthworm digestion systems and the action of related microbes (Ndegwa et al., 2000). It has an excellent ability to improve soil structure, crop productivity, and quality, and thus alleviate crop CCO (Liu et al., 2019; Wang et al., 2021). Humic acid belongs to organic matter originating from the complicated decomposition and transformation processes of plant and animal residues by microorganisms (Li et al., 2019). In addition, the addition of humic acid with inorganic fertilizers can improve soil nutrient content, enhance the fertilizers’ efficiency, accelerate the metabolism of substances in the soil, change the microbial community structure with the increase of beneficial microorganisms, and the reduction of harmful microbiota, and promote crop yield and quality (Chen et al., 2017; Suman et al., 2017; Ahmad et al., 2018; Li et al., 2019). Biochar is a refractory and highly aromatized carbonaceous solid that is produced from the slow thermal degradation of biological biomass under no oxygen or oxygen-deficient conditions (Lehmann and Joseph, 2009), and has the properties of high pH, carbon content, surface area, and large cation adsorption ability (Cantrell et al., 2012; Gul et al., 2015; Yao et al., 2017). It has been widely used in environmental remediation and soil amelioration. For example, Yao et al. (2017) reported that 3 years of biochar amendment changed the soil’s physicochemical properties and might be beneficial in suppressing the growth of crop diseases. Jaiswal et al. (2018) showed that biochar could enhance cucumber growth and reduce damping-off diseases through enriching soil beneficial microbiomes and increasing bacterial and fungal diversity and activity.

The use of a combination of rapeseed cakes and mushroom residues as the primary resources of N/P/K with soil functional amendments (biochar, humic acid, and/or vermicompost) has not recently been reported or applied in tobacco production. Indeed, how FOFs perform in soil and alleviate crop CCO needs to be further deciphered and validated. Returning quantities of biomass residues to the soil is expected to not only achieve the cycle utilization of biomass but also improve soil condition, crop yield, and quality. The objectives of this study are: 1) to study the effects of FOFs on tobacco CCO (primarily on tobacco yield, economic value, and chemical quality); 2) to explore the possibility of using FOFs to replace tobacco-special chemical fertilizers as base fertilizers; and 3) to elucidate the mechanisms of FOFs’ function by paying attention to the changes of rhizosphere soil physicochemical properties, phenolic acids content, and bacterial community structure under field conditions. We assume that FOFs can alleviate tobacco CCO, including the improvement of tobacco yield and chemical quality, by ameliorating the soil’s physicochemical properties and bacterial community structure when compared to CF. Knowledge of FOFs’ efficacy and action mechanisms will provide great potential for the sustainable plantation of tobacco and other crops, and will help to improve soil quality in an environmentally friendly way.

## 2 Materials and methods

### 2.1 Experimental design

The field experiment was conducted from April to September 2020 and was located on Longjie Street, Chengjiang County, Yuxi City, Yunnan Province in China (24°38′39″N, 102°52′33″E). The climate of the study location is as follows: temperature 16–24°C, annual sunshine duration 2, 100–2,300 h; average annual precipitation 837 mm; annual frost-free period 244–365 days; and altitude 1745 m. Tobacco had been continuously cultivated for 2 years in the experimental field before 2020. The tillage regime is the rotation of tobacco and broad bean (*Vicia faba* L.) in the same year. The tobacco variety and soil type are K326 and red soil, respectively. The basic properties of the soil are as follows: pH 7.3, NH_4_^+^-N 7.3 mg kg^−1^, NO_3_^−^-N 8.3 mg kg^−1^, available phosphorous (AP) 37.4 mg kg^−1^, cation exchange capacity (CEC) 20.2 cmol^+^ kg^−1^, organic matter (OM) 26.1 g kg^−1^, total carbon (TC) 26.6 g kg^−1^, total nitrogen (TN) 2.1 g kg^−1^, total phosphorous (TP) 0.9 g kg^−1^, and total potassium (TK) 7.5 g kg^−1^. Tobacco growers usually apply tobacco-special compound chemical fertilizers (total nutrient ≥42%, N: P_2_O_5_: K_2_O = 12: 6: 24) as base fertilizers. We used four types of functional organic fertilizers (FOFs) to displace chemical fertilizers. Rapeseed cakes and mushroom residues are the primary components in the four FOFs, which are added with respective functional substances (i.e., vermicompost, humic acid, and/or wood biochar). Therefore, five fertilizer application programs were included in the study, as follows: CF (tobacco-special chemical fertilizers), VOF (vermicompost-based FOF), HOF (humic acid-based FOF), WOF (wood biochar-based FOF), and COF (compound FOF, vermicompost: humic acid: wood biochar-based FOF = 1: 1: 1). KNO_3_, K_2_SO_4_, (NH_4_)_2_HPO_4_, and CH_4_N_2_O were used to balance the N/P/K content in the FOFs compared to CF. There were four repetitions in each treatment as a randomized block design. Each block owned about 66 m^2^ and was surrounded by guard rows. The CF and FOFs were applied in the planting hole as base fertilizer in April 2020, before transplanting tobacco seedlings at a rate of 225 and 1,000 kg ha^−1^, respectively. Tobacco seedlings were planted with 1.20 × 0.55 m and cultivated according to the local optimal production technology. There were a total of five fertilizer applications during the tobacco planting period—one application of base fertilizer and four applications of topdressing.

### 2.2 Rhizosphere soil and tobacco leaf sampling

In July 2020, we chose four representative tobacco plants of uniform size and dug them up with roots carefully in each block. The non-rhizosphere soil (attached to the root surface loosely) was removed by shaking heavily and the rhizosphere soil (0–5 mm away from the root) of these four tobacco plants was gently collected to form one sample. Immediately, about 50 g of rhizosphere soil per sample was put into an incubator with ice bags, taken back to the laboratory, and put into a –80°C refrigerator for the determination of PA content and bacterial community diversity. The remaining rhizosphere soil was subjected to analysis of the physicochemical properties. Meanwhile, 10 representative tobacco plants in each block were chosen and labeled to determine the agronomic characteristics, including the number of productive leaves, plant height, stem thickness, largest leaf width, and length. The labeled tobacco leaves in each block were separately harvested and flue-cured. After a total of five harvests, we classified the flue-cured tobacco leaves in each block according to the local protocol, weighed each classification, and then calculated economic parameters. The cured leave samples belonging to the C3F classification (i.e., 9th to 14th leaf position, which represents middle leaves) were used to measure chemical components (i.e., nicotine, total sugar, reducing sugar, total nitrogen, potassium, and chlorine) based on the continuous flow method (SEAL AA3, Germany) (Xiao, 1997).

### 2.3 Determination of rhizosphere soil physicochemical properties

The rhizosphere soil was air-dried, ground, and sieved to pass through a 2 mm mesh for analyses of soil pH, NO_3_^−^-N, NH_4_^+^-N, AP, and AK contents and through a 0.15 mm sieve to determine the CEC, OM, total organic matter (TOC), TC, and TN contents. Soil pH was analyzed by a pH parameter (soil: water = 1: 2.5, Multiparameter SevenExcellence, Shanghai, China) (Bao, 2000). Soil NO_3_^−^-N and NH_4_^+^-N were extracted in potassium chloride solution and determined by an ultraviolet spectrophotometer (UV-1890, Daojin Instrument Co., Ltd., Jiangsu, China; Ministry of Agriculture of the People’s Republic of China, GB/T 32737-2016, 2016; Ministry of Environmental Protection of the People’s Republic of China, HJ 634-2012, 2012). AP in acid and alkaline soil was extracted in HCl-H_2_SO_4_ and NaHCO_3_ solution, respectively, and then determined by an ultraviolet spectrophotometer (UV-1890, Daojin Instrument Co., Ltd., Jiangsu, China) (Bao, 2000). Soil AK was extracted in ammonium acetate solution and evaluated by an atomic absorption spectrometer (AAS, Analytik Jena novAA 300, Germany; Bao, 2000). Soil extraction in hexammine cobalt trichloride solution was used to analyze CEC by a microplate reader (BioTek Epoch2, United States; Ministry of Environmental Protection of the People’s Republic of China, HJ 889-2017, 2017). The TOC and OM contents were determined through the potassium dichromate-sulfuric acid method, and the TC and TN contents in the soil were measured by an elemental analyzer (Elemental Vario EL Cube, Germany) (Bao, 2000).

### 2.4 Determination of phenolic acid content in rhizosphere soil

The method to determine the PA content was drawn from Tan et al. (2008) with little change. Moist rhizosphere soil (15 g) was set overnight with 15 ml of 1 M NaOH and was then shaken at 210 rpm at 25°C for 30 min the next day. The suspension was centrifuged at 8,000 × *g* for 10 min 10 ml of supernate was acidified with 12 M HCl to pH 2.5 and was then put for 2 h for humic acid precipitation. After that, the suspension was centrifuged at 8,000 × *g* for 10 min and the supernate was passed through a 0.22 μm organic filter subjected to UPLC (Agilent 1,290, Agilent Technologies Inc., United States). The UPLC analytical conditions for PA were as follows: chromatographic column, C_18_ (CAPCELL PAK MGⅡ, 4.6 × 250 mm); column temperature, 40°C; detector wavelength, 280 nm; flow velocity, 1 ml min^−1^; and injection volume, 10 μL. The mobile phase consisted of 0.1% phosphoric acid solution (A-phase) and acetonitrile (B-phase). In total, 17 types of standard PA samples (i.e., gallic acid, phthalic acid, *p*-hydroxybenzoic acid, caffeic acid, vanillic acid, vanillin, benzoic acid, coumalic, salicylic acid, ferulic acid, sinapic acid, benzothiazole, trans-cinnamic acid, diethy phthalate, benzyl benzoate, 4-methylphenyl benzoate, and syringic acid) were measured for the retention time and peak size under a certain concentration. The PA kinds and concentrations in rhizosphere soil were identified by comparing retention time and peak size with respective standards.

### 2.5 Bacterial community diversity analysis in rhizosphere soil

#### 2.5.1 DNA extraction and PCR amplification

According to the manufacturer’s protocol, the microbial community genomic DNA of rhizosphere soil was extracted with the instructions of Fast DNA^®^ Spin Kit for Soil (MP Biomedicals, LLC 29525 Fountain Parkway, Solon, OH, 44139 United States). The purity and concentration of DNA were determined based on the 260/280 and 260/230 nm ratios through a micro-spectrophotometer (Nano-300, Allsheng, Hangzhou, China). DNA integrity was determined by 1.0% agarose gel electrophoresis and visualized. The hypervariable region V3-V4 of the bacterial 16S rRNA gene was amplified using a barcode sequences primer pair 338F (5′-ACT​CCT​ACG​GGA​GGC​AGC​AG-3′) and 806R (5′-GGACTACHVGGGTWTCTAAT-3′). The PCR reactions were performed in 20 uL reaction mixtures: 5 × *TransStart* FastPfu buffer 4 μL, 2.5 mM dNTPs 2 μL, 5 uM forward primer 0.8 μL, 5 uM reverse primer 0.8 μL, *TransStart* FastPfu DNA Polymerase 0.4 μL, BSA 0.2 μL, template DNA 10 ng, and finally ddH_2_O up to 20 uL. The PCR thermal cycling conditions were performed as the regime: initial denaturation (95°C for 3 min), followed by 27 cycles of denaturation (95°C for 30 s), annealing (55°C for 30 s), extension (72°C for 45 s), and a final extension (72°C for 10 min). Each sample was amplified in triplicate. PCR product was extracted from 2% agarose gels, purified using the AxyPrep DNA Gel Extraction Kit (Axygen Biosciences, Union City, CA, United States) according to manufacturer’s instructions, and quantified using Quantus™ Fluorometer (Promega, United States).

#### 2.5.2 Illumina MiSeq sequencing

The purified amplicons were pooled in equimolar and paired-end sequenced on an Illumina MiSeq PE300 platform (Illumina, San Diego, United States) by Majorbio Bio-Pharm Technology Co. Ltd. (Shanghai, China).

#### 2.5.3 Processing of sequencing data

The raw 16S rRNA gene sequencing reads were demultiplexed and quality-filtered by fastp v0.19.6 (Chen et al., 2018a). Pair-end sequences were assembled by FLASH v1.2.11 (Magoč and Salzberg, 2011). Bacterial community operational taxonomic units (OTUs) with a 97% similarity cutoff were clustered by UPARSE v7.0.1090 (Stackebrandt and Goebel, 1994; Edgar, 2013), and chimeric sequences were identified and removed. The taxonomy affiliation of each OTU representative sequence was carried out through RDP Classifier v2.11 (Wang et al., 2007) against the silva138/16S rRNA database (https://www.arb-silva.de/) with a classification confidence of 0.7.

#### 2.5.4 Statistical analysis

Except for the microbial data, the data were analyzed by SPSS 26.0. One-way ANOVA analysis of variance followed by Duncan’s test was carried out at the level of significance (*p* < 0.05). OTUs counts were normalized based on the minimum in all samples before bioinformatic analysis. All of the sequences were processed using QIIME v1.9.1 (Caporaso et al., 2010). The alpha-diversity (α-diversity) of the bacterial community was determined, including the ACE, Chao, Shannon, and Simpson indicators using Mothur v1.30.2 (Schloss et al., 2009). Petaline diagrams were constructed to show the number of OTUs exclusive and shared among treatments. For the beta-diversity (β-diversity) of microbial communities, we used permutational multivariate analysis of variance (PERMANOVA) to determine the significance of Bray-Curtis principal coordinate analysis (PCoA). The significance of Bray-Curtis non-metric multidimensional scaling (nMDS) was measured by the Analysis of similarities (ANOSIM) test. We discovered the biomarkers among five treatments using the linear discriminant analysis effect size (LEfSe) tool (Segata et al., 2011). The correlation between the relative abundance of microbial communities and soil environmental factors was analyzed by correlation heatmap and redundancy analysis (RDA). PCoA, nMDS, LEfSe, correlation heatmap, and RDA were performed by R v3.1.1 (Li et al., 2019; Liu et al., 2020). PERMANOVA and ANOSIM tests were achieved by QIIME v1.9.1 (Caporaso et al., 2010).

## 3 Results

### 3.1 The tobacco agronomic characteristics, economic, and quality parameters were improved under FOF treatments

**FIGURE 1:**
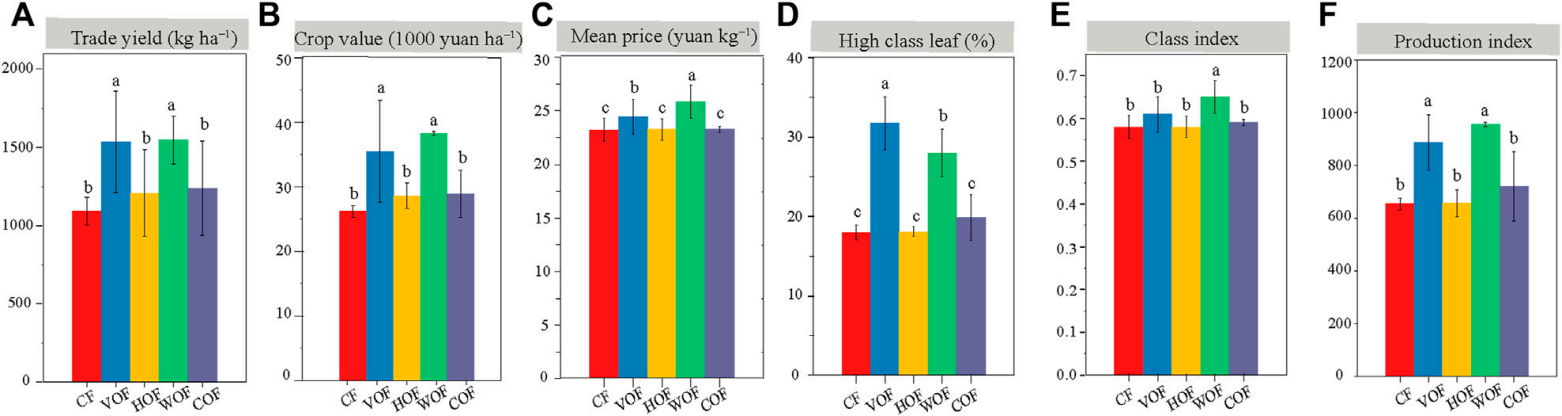
Yield **(A)** and economic value **(B–F)** of tobacco leaf. CF, VOF, HOF, WOF, and COF represent the treatments of tobacco-special chemical fertilizer, vermicompost-based, humic acid-based, wood biochar-based, and compound functional organic fertilizers, respectively. Different letters within each index show a significant difference based on the Duncan’s method (*p* < 0.05).

The tobacco agronomic characteristics in the four FOFs treatment groups were all better than CF, including plant height, stem girth, maximum leaf area, and the number of productive leaves (Supplementary Figure S1). There was no evident difference in tobacco agronomic traits among four FOFs. The trade yield and value of tobacco leaf in VOF (1,537 kg ha^−1^ and 35,000 yuan ha^−1^) and WOF (1,547 kg ha^−1^ and 38,000 yuan ha^−1^) increased apparently (*p* < 0.05) than CF (1,093 kg ha^−1^ and 26,000 yuan ha^−1^) (Figures 1A,B). However, they did not present a significant difference (*p* ≥ 0.05) among HOF (1,083 kg ha^−1^ and 26,000 yuan ha^−1^), COF (1,114 kg ha^−1^ and 26,000 yuan ha^−1^), and CF. The tobacco yield and value increased by 40.6% and 35.6% in VOF, and by 41.5% and 46.1% in WOF than those in CF, respectively. Simultaneously, the tobacco leaf mean price, high class leaf rate, class index, and production index in FOFs were all higher than those in CF, with VOF and WOF presenting extraordinary enhancements (Figures 1C–F). The tobacco’s chemical quality indicators are shown in Table 1 and almost all met the high-quality tobacco standard according to local protocols. The nicotine, TN, and Cl content decreased, and the total sugar, reducing sugar, and K_2_O content increased in FOFs when compared with CF. Overall, the tobacco quality was improved under FOFs, with higher values of total sugar/nicotine, K_2_O/Cl, reducing sugar/nicotine, and a lower value of Cl/nicotine than CF.

**TABLE 1:** Tobacco leaf quality parameters (mean ± SD). CF, VOF, HOF, WOF, and COF represent the treatments of tobacco-special chemical fertilizer, vermicompost-based, humic acid-based, wood biochar-based, and compound functional organic fertilizers, respectively. Different letters show a significant difference within each column based on Duncan’s method (*p* < 0.05).

	Nicotine (%)	Total sugar (%)	Reducing sugar (%)	Total nitrogen (%)	K_2_O (%)	Cl (%)	Total sugar/nicotine	Reducing sugar/nicotine	Total nitrogen/nicotine	K_2_O/Cl
CF	2.48 ± 0.16 a	25.8 ± 2.5 b	20.1 ± 1.5 c	1.88 ± 0.08 a	2.07 ± 0.00 b	0.70 ± 0.14 a	10.5 ± 1.6 b	7.6 ± 0.2 b	0.76 ± 0.05 a	2.93 ± 0.05 c
VOF	2.31 ± 0.19 b	31.8 ± 3.1 a	25.0 ± 2.6 a	1.70 ± 0.09 b	2.14 ± 0.11 ab	0.47 ± 0.08 b	13.8 ± 2.1 a	10.9 ± 1.7 a	0.74 ± 0.09 a	4.51 ± 0.17 a
HOF	2.42 ± 0.10 ab	29.5 ± 2.9 ab	23.5 ± 2.9 ab	1.82 ± 0.32 ab	2.17 ± 0.29 ab	0.67 ± 0.04 a	12.3 ± 0.7 ab	9.8 ± 0.8 ab	0.69 ± 0.10 a	3.23 ± 0.71 b
WOF	2.41 ± 0.04 ab	28.9 ± 3.3ab	23.4 ± 3.2 ab	1.76 ± 0.14 ab	2.27 ± 0.05 a	0.69 ± 0.12 a	12.0 ± 1.4 ab	9.7 ± 1.4 ab	0.73 ± 0.05 a	3.29 ± 0.63 b
COF	2.33 ± 0.13 b	28.9 ± 2.0 ab	21.7 ± 2.0 bc	1.71 ± 0.14 b	2.17 ± 0.18 ab	0.64 ± 0.18 a	12.7 ± 1.0 ab	9.8 ± 0.9 ab	0.71 ± 0.03 a	3.39 ± 0.39 b

### 3.2 Rhizosphere soil physicochemical properties were ameliorated and phenolic acid content decreased in FOF treatments

Rhizosphere Soil pH showed an acidification trend after planting tobacco, which changed from 7.34 to 6.73–6.94 (Table 2). This acidification trend was mitigated under the functions of VOF and WOF. The soil’s NO_3_^−^-N and TN content did not show any obvious variance under the five treatments (*p* ≥ 0.05). Although the OM, TOC, and TC contents increased in VOF and HOF, they did not present a significant difference (*p* ≥ 0.05). Meanwhile, the FOFs had a greater effect on the NH_4_^+^-N, AP, AK contents, and CEC, particularly on the AP content. The NH_4_^+^-N, AK contents, and CEC showed an increasing trend under FOFs, except for COF, when compared with CF. The AP content increased by 31.6–81.0% in the four FOFs treatments when compared to CF.

**TABLE 2:** Soil physicochemical properties when sampling tobacco (mean ± SD). CF, VOF, HOF, WOF, and COF represent the treatments of tobacco-special chemical fertilizer, vermicompost-based, humic acid-based, wood biochar-based, and compound functional organic fertilizers, respectively. Different letters within each column show a significant difference in soil physicochemical properties based on Duncan’s method (*p* < 0.05).

	pH	NO_3_^−^-N (mg kg^−1^)	NH_4_^+^-N (mg kg^−1^)	Available P (mg kg^−1^)	Available K (mg kg^−1^)	CEC (cmol^+^ kg^−1^)	OM (g kg^−1^)	TOC (g kg^−1^)	TC (%)	TN (%)
CF	6.73 ± 0.09 b	4.37 ± 1.71 a	3.04 ± 0.02 b	53.2 ± 13.0 c	342.7 ± 18.3 b	19.6 ± 1.5 ab	32.9 ± 4.1 a	19.1 ± 2.4 a	3.18 ± 0.47 ab	0.22 ± 0.02 a
VOF	6.80 ± 0.19 b	4.46 ± 0.10 a	4.82 ± 1.02 ab	94.4 ± 17.0 a	410.9 ± 42.5 a	20.2 ± 0.8 ab	34.9 ± 4.7 a	20.2 ± 2.7 a	3.23 ± 0.40 ab	0.23 ± 0.01 a
HOF	6.74 ± 0.15 b	4.19 ± 1.28 a	5.32 ± 1.04 a	96.3 ± 12.3 a	373.9 ± 2.0 ab	20.1 ± 0.9 ab	36.3 ± 4.6 a	21.0 ± 2.7 a	3.34 ± 0.39 a	0.23 ± 0.02 a
WOF	6.94 ± 0.18 a	4.10 ± 1.01 a	3.32 ± 1.06 b	84.1 ± 28.3 ab	369.3 ± 27.2 ab	20.8 ± 0.8 b	34.9 ± 6.6 a	20.3 ± 3.8 a	3.07 ± 0.13 ab	0.22 ± 0.01 a
COF	6.75 ± 0.04 b	3.37 ± 0.89 a	2.97 ± 0.23 b	70.0 ± 6.6 bc	332.6 ± 35.7 b	17.8 ± 2.6 a	30.5 ± 4.0 a	17.7 ± 2.3 a	2.95 ± 0.37 b	0.22 ± 0.02 a

**FIGURE 2:**
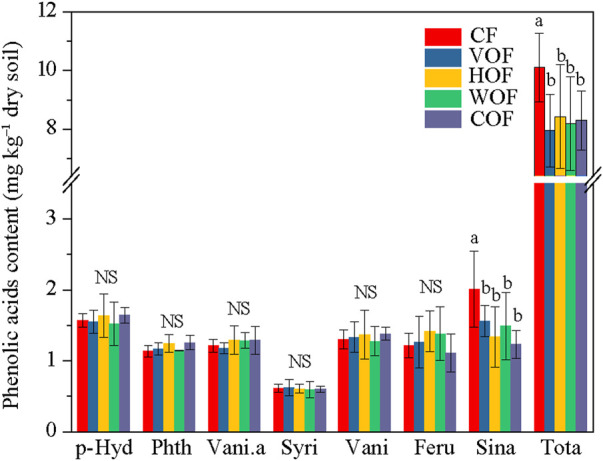
Phenolic acid content in the rhizosphere soil. CF, VOF, HOF, WOF, and COF represent treatments of tobacco-special chemical fertilizer, vermicompost-based, humic acid-based, wood biochar-based, and compound functional organic fertilizers, respectively. P-Hyd, Phth, Vani.a, Syri, Vani, Feru, Sina, and Tota represent *p*-hydroxybenzoic acid, phthalic acid, vanillic acid, syringic acid, vanillin, ferulic acid, sinapic acid, and total phenolic acids, respectively. Different letters show a significant difference among the five treatments based on the Duncan’s method (*p* < 0.05), and NS indicates no significant difference (*p* ≥ 0.05).

Although we prepared 17 varieties of the PA’s standard reference samples, only seven types (*p*-hydroxybenzoic acid, phthalic acid, vanillic acid, syringic acid, vanillin, ferulic acid, and sinapic acid) could be detected in the rhizosphere soil (Figure 2). The *p*-hydroxybenzoic acid average content was the highest (1.52–1.64 mg kg^−1^ dry soil), followed by the sinapic acid (1.23–2.01 mg kg^−1^ dry soil), vanillin (1.28–1.38 mg kg^−1^ dry soil), ferulic acid (1.11–1.42 mg kg^−1^ dry soil), vanillic acid (1.18–1.29 mg kg^−1^ dry soil), and phthalic acid contents (1.14–1.26 mg kg^−1^ dry soil). The syringic acid content was the lowest (0.60–0.62 mg kg^−1^ dry soil). The total PA content ranged from 7.95 to 10.10 mg kg^−1^ dry soil in all treatments. Although the sinapic acid and total PA contents decreased apparently under FOFs when compared to CF (*p* < 0.05), they did not show an obvious variance among the four FOF treatments. There was no significant difference (*p* ≥ 0.05) for the other PAs among the five treatments.

### 3.3 Bacterial community diversities in rhizosphere soil were changed under the different treatments

**FIGURE 3:**
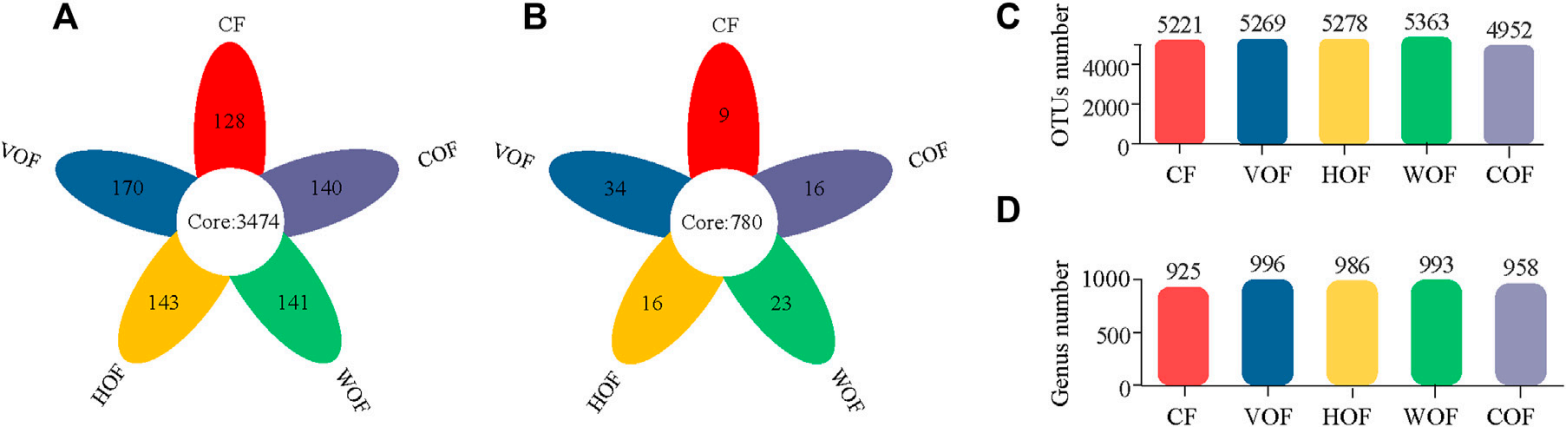
Shared, unique, and total numbers of bacterial OTUs **(A,C)** and genera **(B,D)** in the rhizosphere soil among the five treatments. CF, VOF, HOF, WOF, and COF represent the treatments of tobacco-special chemical fertilizer, vermicompost-based, humic acid-based, wood biochar-based, and compound functional organic fertilizers, respectively.

**FIGURE 4:**
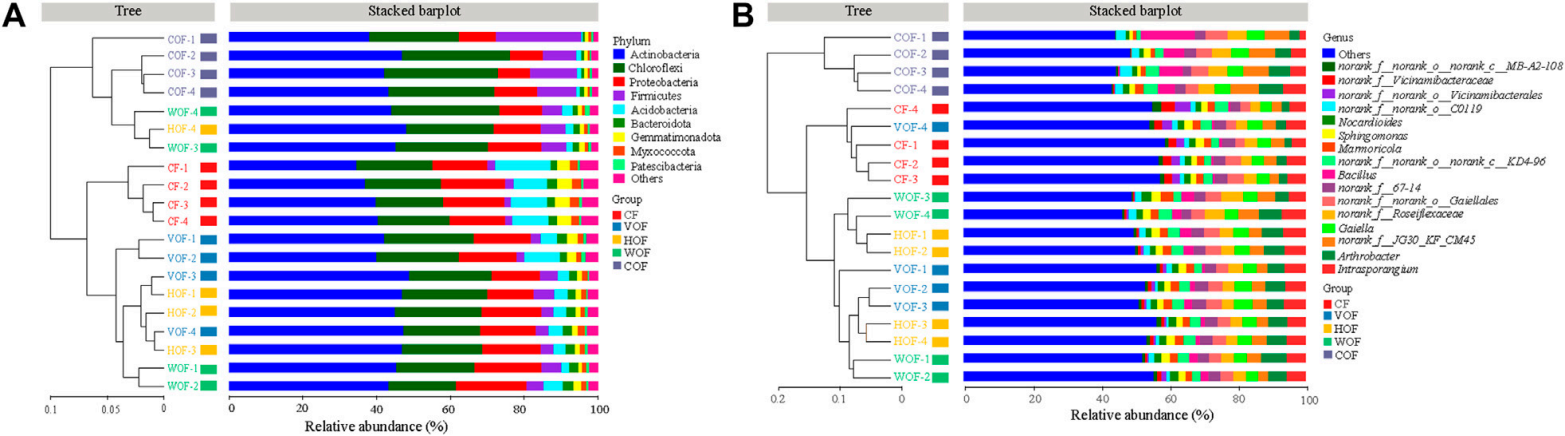
Hierarchical clustering tree of samples in bacterial community at the phylum **(A)** and genus **(B)** level in the rhizosphere soil. The horizontal histogram shows the species composition in top nine phylum **(A)** and top 16 genus **(B)** in different treatments. CF, VOF, HOF, WOF, and COF represent the treatments of tobacco-special chemical fertilizer, vermicompost-based, humic acid-based, wood biochar-based, and compound functional organic fertilizers, respectively.

A total of 2,092,458 sequences with an average length of 414 bp were produced after quality-controlled matching. The Coverage index of all samples was >97.2% and the rarefaction curve tended to reach the saturation point, which indicates that the sequencing depth was able to reflect the microbial community in our study (Supplementary Figure S2). We gained 6,876 OTUs in all samples. There were 3,474 bacterial OTUs shared among the five treatments, and 128, 170, 143, 141, and 140 OTUs were unique in CF, VOF, HOF, WOF, and COF treatments, respectively (Figure 3A). There were more OTUs numbers in VOF, HOF, and WOF when compared to CF, with WOF having the most OTUs with 5,363 (Figure 3C). However, T4 had the least OTUs with 4,952. Meanwhile, there were 780 genera shared by five treatments, and 9, 34, 16, 23, and 16 genera were unique for CF, VOF, HOF, WOF, and COF, respectively (Figure 3B). The genus types increased to 958–996 in four FOFs compared to 925 in CF (Figure 3D). The first five highest relative abundance of bacteria at the phylum level were *Actinobacteria* (40.2–43.8% in all samples), *Chloroflexi* (20.6–25.0%), *Proteobacteria* (13.5–16.4%), *Acidobacteria* (1.8–7.7%), and *Firmicutes* (3.0–9.1%), with an average total relative abundance of 90.0% (Figure 4A). The relative abundance of *Actinobacteria* and *Firmicutes* increased, and *Acidobacteria* and *Gemmatimonadota* declined in the four FOF treatments when compared to CF. The first five highest relative abundance of bacteria at the genus level were *Intrasporangium* (4.3–5.9% in all samples), *Arthrobacte*r (4.1–5.7%), *norank_f__Roseiflexaceae* (3.7–5.5%), *norank_f__JG30-KF-CM45* (3.6–5.6%), and *Gaiella* (3.4–4.5%), with the average total relative abundance of 22.5% (Figure 4B). The distribution pattern of the bacterial community at the genus level in COF differentiated from the other four treatments, with an apparent increase of *bacillus* in COF.

**FIGURE 5:**
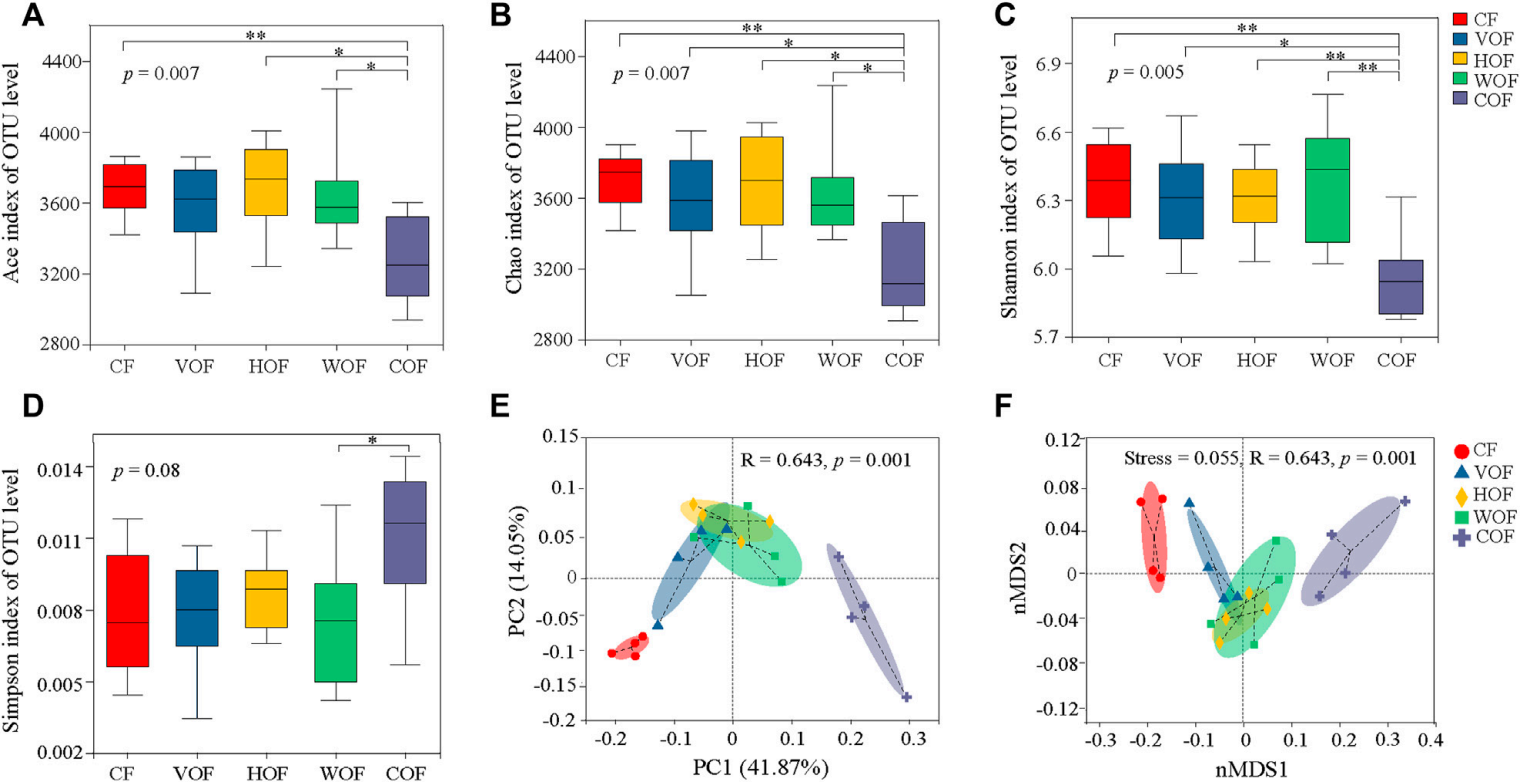
Effect of different fertilizers on bacterial community structure in the rhizosphere soil. Ace index **(A)**, Chao index **(B)**, Shannon index **(C)**, and Simpson index **(D)** of bacteria. * and ** indicate statistical significance at *p* < 0.05 and *p* < 0.01 based on the Wilcoxon rank-sum test, respectively. Principal coordinate analysis (PCoA) **(E)** and non-metric multidimensional scaling (nMDS) **(F)** of bacterial community on OTUs level based on Bray-Curtis distance. CF, VOF, HOF, WOF, and COF represent the treatments of tobacco-special chemical fertilizer, vermicompost-based, humic acid-based, wood biochar-based, and compound functional organic fertilizers, respectively.

For the α-diversity of the bacterial community on the OTUs level, COF had the minimum of the Ace, Chao, and Shannon indexes, and the maximum of the Simpson index. This indicates that the α-diversity in COF was the lowest in all treatments (Figures 5A–D). Moreover, the α-diversity indexes in COF were significantly (*p* < 0.05) different from the other four treatments, and there was no significant difference (*p* ≥ 0.05) among CF, VOF, HOF, and WOF. Based on PCoA and PerMANOVA analyses on the OTUs level, the five treatments were divided from each other, particularly at the first primary component, with the first two primary components explaining 41.87% and 14.05% variance, respectively (R = 0.643, *p* = 0.001) (Figure 5E). This phenomenon was also revealed from Bray-Curtis nMDS and ANOSIM analyses (Figure 5F), which showed remarkable variations in the β-diversity of bacterial community among the five treatments (stress = 0.055, R = 0.643, *p* = 0.001). CF was prominently distinct from COF, and VOF, HOF, and WOF clustered relatively closer, presenting a more pronounced difference in the bacterial community’s β-diversity between CF and COF than that among VOF, HOF, and WOF.

**FIGURE 6:**
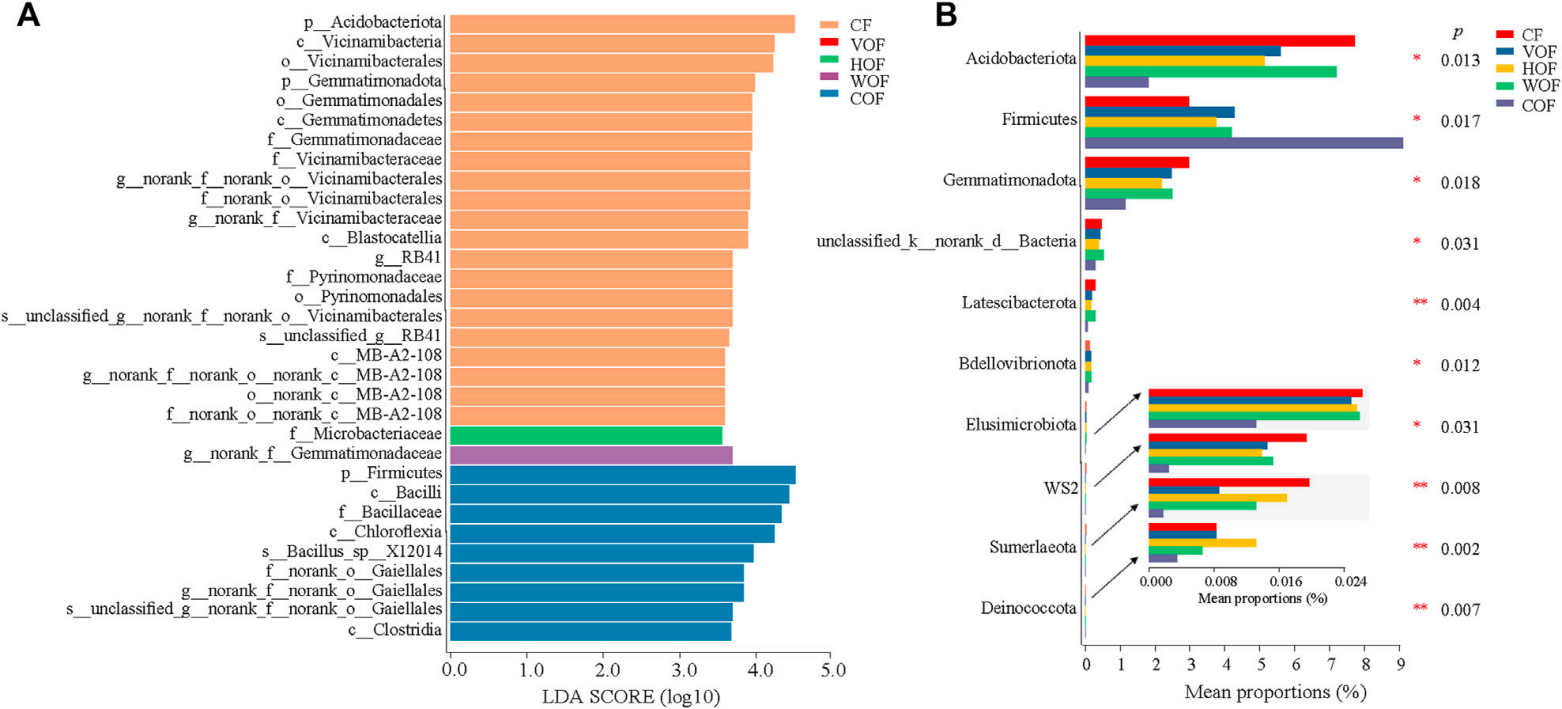
LEfSe **(A)** and comparative analyses **(B)** of bacterial community at the phylum level in the rhizosphere soil based on the Kruskal–Wallis H test. CF, VOF, HOF, WOF, and COF represent the treatments of tobacco-special chemical fertilizer, vermicompost-based, humic acid-based, wood biochar-based, and compound functional organic fertilizers, respectively. Only the top 10 bacteria that showed a significant difference (*p* < 0.05) among the five treatments are shown.

Based on the LEfSe analysis, every group except VOF extinguished some biomarkers contributing to group differences, and CF and COF had more varieties of biomarkers (Figure 6A; Supplementary Figure S3). This shows that the most important biomarkers per group were phylum-*Acidobacteriota* in CF, family-*Microbacteriaceae* in HOF, genus-*norank_f__Gemmatimonadaceae* in WOF, and phylum-*Firmicutes* in COF. From the significance analysis for the bacterial community at the phylum level among groups based on the Kruskal–Wallis H test, there was a significant increase in the relative abundance of *Firmicutes*, and a significant decrease of *Acidobacteriota*, *Gemmatimonadota, Elusimicrobiota, WS2* and *Sumerlaeota* in the four FOF treatments compared to CF, particularly in COF (Figure 6B). The relative abundance of *unclassified_k__norank_d__Bacteria, Latescibacterota, Bdellovibrionota*, and *Deinococcota* declined remarkably in COF when compared to the other four treatments. Meanwhile, the relative abundance of some bacterial genus-*Streptomyces, Bacillus*, *Arthrobacter,* and *Paenibacillus* increased in the four FOF treatments, without a significant difference among the five treatments (Supplementary Figure S4).

**FIGURE 7:**
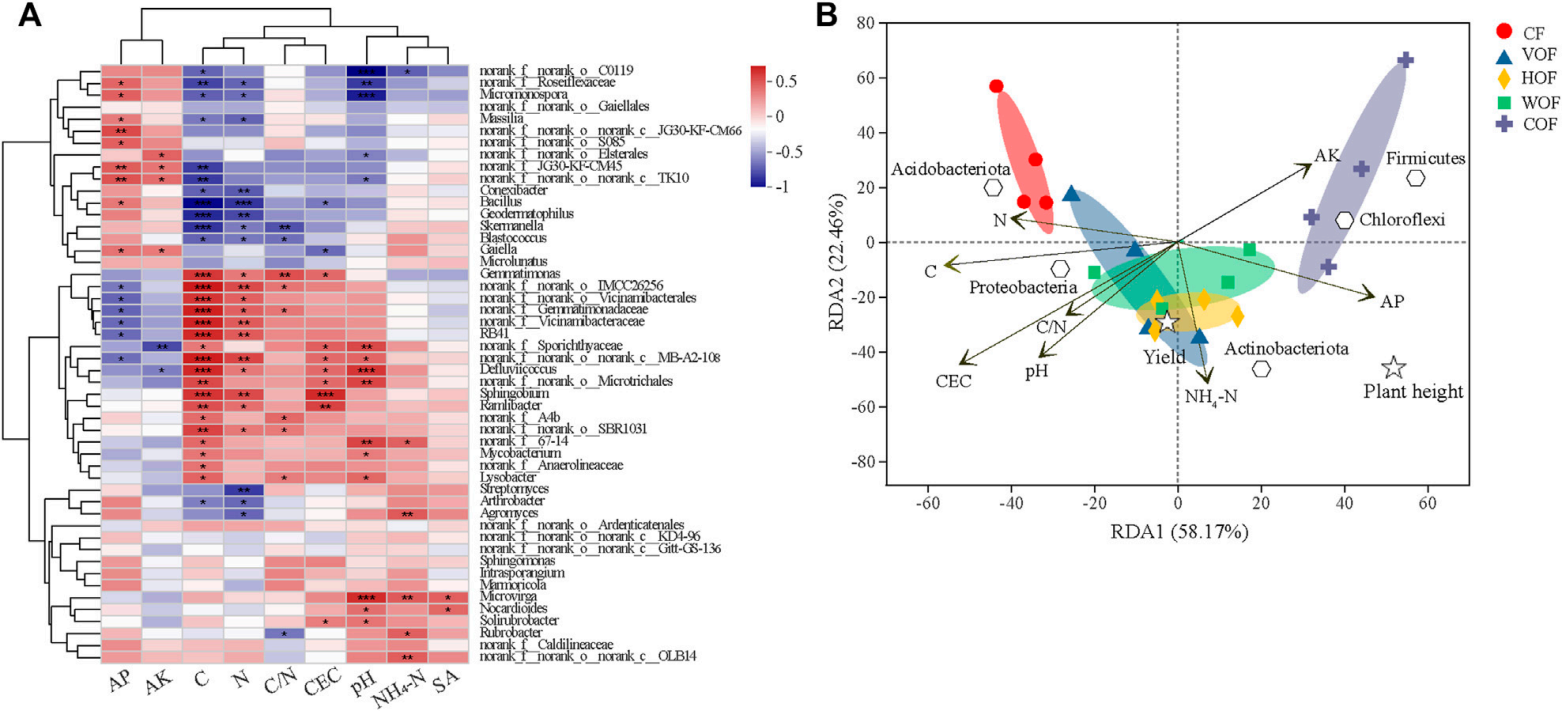
Spearman correlation heatmap between bacterial genera and soil environmental factors **(A)** and RDA of the relationship among bacterial phylum, samples, environmental variables, tobacco height, and leaf yield **(B)**. *, **, and *** indicate statistical significance at *p* < 0.05, *p* < 0.01, and *p* < 0.001, respectively. AP, AK, and SA represent the soil available P, available K, and sinapic acid contents. CF, VOF, HOF, WOF, and COF represent the treatments of tobacco-special chemical fertilizer, vermicompost-based, humic acid-based, wood biochar-based, and compound functional organic fertilizers, respectively.

From the correlation heatmap between the bacterial community at the genus level and rhizosphere soil environmental factors, soil TC and TN were the most important factors that influence the bacterial composition, which showed an intimately similar relationship (*p* < 0.05) with more genera (Figure 7A). The relationship pattern between soil bacteria composition and TC, TN, C/N, and CEC clustered together, which is the opposite of that between soil bacteria and AP, AK. The first two predominant components explained 58.17% and 22.46% (80.63% in total) of the total variance based on the RDA analysis, respectively (Figure 7B). The four samples in the same group clustered together, and different groups separated primarily at the first primary component. Soil TC, TN, CEC, pH, NH_4_^+^-N, AP, and AK were the most crucial environmental factors to influence the bacterial composition. Soil TC and TN had a positive effect, while AK and AP had a negative influence on the bacterial community in CF, which was the opposite in COF. Simultaneously, there was a positive relationship between soil NH_4_^+^-N content and the bacterial community in HOF and WOF. Tobacco plant height was positively correlated with soil NH_4_^+^-N, AK, AP content, and the relative abundance of *Actinobacreriota*, *Chloroflexi*, and *Firmicutes*. The tobacco leaf yield, soil NH_4_^+^-N, TC content, and the relative abundance of *Proteobacteria* and *Actinobacteriota* were all promoted.

## 4 Discussion

### 4.1 Effects of different fertilizers on tobacco agronomic traits, leaf yield, value, and chemical quality

The tobacco agronomic traits, tobacco leaf trade yield, value, and chemical quality were improved in FOF treatments when compared to CF. The tobacco plant agronomic traits showed no apparent difference in the four FOF treatments. This indicates that FOFs could promote tobacco plant growth and that this promotion level was similar under different types of FOFs in our study. There was an evident increase in tobacco leaf trade yield and value in VOF and WOF, while no significant difference was observed among CF, HOF, and COF. Vermicompost-based and wood biochar-based FOFs promoted the enrichment of tobacco leaf biomass and the formation of high-quality tobacco leaf. However, humic acid-based and compound FOFs did not present such a phenomenon. Vermicompost performs well with high porosity, aeration, and water-holding capacities, and has abundant nutrient elements, plant growth-promoting substances, and beneficial antagonistic microbiomes, which is typical of organic fertilizers and soil conditioners (Edwards and Neuhauser, 1988; Wang et al., 2021). It can alleviate crop CCO, and improve crop yield and quality by enhancing soil physicochemical properties and microbial community structure under field conditions (Liu et al., 2019; Wang et al., 2021). Biochar is well-known to be an excellent soil ameliorant that can alleviate soil acidification; increase soil TC and nutrient contents; improve soil structure, nutrient-holding, water-holding capacities, and enzyme activities; and change the structure of the soil’s microbial community (Lehmann et al., 2011). Biochar can promote the abundance and activities of beneficial microbes by providing a good habitat for them (Palansooriya et al., 2019). It has also been verified that vermicompost and biochar can improve soil properties, and can improve the yield and quality of continuous cropping cucumber for different years (Wang et al., 2021). These two FOFs can provide tobacco plants with effective nutrients and a more suitable soil micro-ecology condition, which then contribute to the growth of tobacco. Although the positive effect of humic acid fertilizer on peanut yield has been verified based on a 3-year experiment (Li et al., 2019), it was not suitable to apply to continuous cropping tobacco in the recent study. The reason for this is that humic acid accelerates soil acidification, which is an important factor causing K326 CCO (Bai et al., 2019). Meanwhile, soil acidification in red soil is prevalent and relentless owing to the soil erosion processes, the acid atmospheric deposition, and the abuse application of chemical fertilizers. Therefore, strong acid HOF is not advisable to apply in red soil planting K326 on the basis of our results. Intriguingly, the compound FOF did not present a promotion influence on tobacco leaf yield, which could be due to an antagonistic effect among the components or to the strong influence of humic acid. Which components show an antagonistic effect and how they cooperate were not clear in the compound FOF in this study, and this needs to be further researched.

The chemical quality of tobacco leaf (total sugar/nicotine, reducing sugar/nicotine, Cl/nicotine, K_2_O/Cl) was improved in the four FOF treatments. Similarly, no apparent difference in tobacco leaf quality was discovered among the four FOF treatments. The enhancement of organic fertilizers on plant yield and quality has been demonstrated in many studies (Liu et al., 2018; Yan and Liu, 2020; Chen D et al., 2022; Imran et al., 2022). Organic additives can suppress tomato disease and improve the yield compared to chemical fertilizers (Liu et al., 2018). Furthermore, Yan and Liu (2020) verified that high-carbon fertilizers could increase tobacco quality mainly by improving the soil carbon pool. The soil TOC, OM, NH_4_^+^-N, AP, and AK contents increased in VOF, HOF, and WOF, which could provide tobacco with more nutrients, enhance soil ventilation and aggregation environment, and thus stimulate tobacco growth. Particularly, the soil AP content showed a marked increase in all four FOF treatments. The scarcity of P element in red soil is a prevalent issue in Southern China, and it has been reported that peanut yield could be promoted as soil P status increased (Chen et al., 2018b). The application of FOFs provides an underlying effective strategy in alleviating P scarcity in broad red soil. Organic fertilizers can improve the soil OM, NH_4_^+^-N, AP, and AK contents when compared to chemical fertilizers, which has been verified in other studies (Liu et al., 2018; Li et al., 2019; Wang et al., 2021). Additionally, low pH can promote the occurrence of the pathogenic bacteria *Ralstonia solanacearum*, which attacks tobacco roots (Li et al., 2017). There was a possibility that FOFs inhibited the growth and colonization of underlying pathogens by improving soil pH.

### 4.2 Effects of different fertilizers on phenolic acid content in the rhizosphere soil

Different plants release their unique varieties of PA. Meanwhile, *P*-hydroxybenzoic acid, phthalic acid, vanillic acid, syringic acid, vanillin, ferulic acid, and sinapic acid were the main types of PA in the rhizosphere soil of K326, which was almost in accordance with the results of Chen L. M et al. (2022). The sinapic acid and total PA contents in rhizosphere soil decreased apparently (*p* < 0.05) in the four FOF treatments, and no significant difference (*p* ≥ 0.05) was observed for the other PAs, which are a class of small organic substances and secondary metabolites that are released from plants and have strong allelopathy (Hiraddate et al., 2005). PAs can be enriched after continuous cropping of the same plants in the soil through plant evaporation, leaching, root secretion, and the degradation of litter and residues over the years (Weir et al., 2004). After accumulation to a specific concentration, PAs play a crucial negative role in seed germination, plant antioxidant system, cell structure, and the respiration and growth of plant roots, and can ultimately destroy the plant’s normal growth (Wu and Ma, 2006). Meanwhile, there is an intimate correlation between PA and microbiota in soil. As a result, the allelopathy of PA is considered to be one of the main causes of tobacco CCO (Chen et al., 2018a; Bai et al., 2019). This allelopathy function was weakened under the function of FOFs, which may have contributed to the improvement of tobacco yield and quality in the recent study. Moreover, there was a positive relationship between the content of sinapic acid, and the relative abundance of *Microvirga* and *Nocardioides* (*p* ≤ 0.05) in the rhizosphere soil based on the Spearman correlation analysis. Therefore, PA has changed the bacterial structure and diversity to some extent.

### 4.3 Effects of different fertilizers on the bacterial community structure and diversity in the rhizosphere soil

Our results show that the bacterial structure and diversity in rhizosphere soil were changed under the function of the FOFs. Different fertilizer management programs can significantly change the soil’s microbial community, biomass, and diversity, and functional activities can be enhanced under balanced fertilization (Liu et al., 2018; Li et al., 2019; Liu et al., 2020). There were 3,474 OTUs and 780 genera shared among the five treatments from the petaline diagrams, which indicates that the major bacterial species co-existed in different treatments. There were more OTUs in the FOF treatments, except for COF, and more genera in all four FOFs treatments compared to CF. This shows that FOFs improved the rhizospheric bacterial richness. These results are similar to previous studies where there were more bacterial populations in organic-treated soils than in chemical fertilizers treatment (Witter et al., 1993). Wang et al. (2021) also found that biochar and vermicompost could increase soil bacterial numbers. From the α-diversity analysis of bacteria at the OTU level, no significant difference for Ace, Chao, Shannon, and Simpson indexes was observed in CF, VOF, HOF, and WOF. However, these four indexes in COF were obviously distinct from the other four treatments. COF obviously declined bacterial α-diversity. This may be related to an antagonism reaction among the components in COF. From the β-diversity analysis of bacteria at the OTU level based on PCoA and nMDS, the samples in the same fertilization management program were grouped more closely, while those in distinct programs were well separated from each other along with the first component, especially for CF and COF. This indicates that FOFs changed the bacterial community structure compared with CF, with a more apparent change in COF. Other studies have reported that an application of organic additives apparently shifted the soil’s microbial community (Bonanomi et al., 2010; Liu et al., 2018), which is in accordance with our results. The bacterial community structure did not distinctly vary from each other in VOF, HOF, and WOF. This happened because these three types of FOFs shared some of the same components. Meanwhile, the time interval between the application of FOFs and sampling maybe did not reach the point where it could cause a significant variance.

*Actinobacteria*, *Chloroflexi*, *Proteobacteria*, *Acidobacteria*, *Firmicutes*, and *Gemmatimonadota* were the most abundant bacterial phyla in the rhizosphere soil in our study, which is generally consistent with previous studies (Li et al., 2019; Liu et al., 2020). *Actinobacteria* and *Firmicutes* showed an increasing trend, while *Acidobacteria* and *Gemmatimonadota* showed a declining trend in the relative abundance in four of the FOF treatments. Wu et al. (2014) reported that *Actinobacteria* was negatively associated with tobacco bacterial wilt disease rate (*r* = –0.728) and was found in more abundance in organic fertilizer treatment. Li et al. (2019) also found that the abundance of *Firmicutes* increased in peanut-planting soil with the application of humic acid fertilizer, which has been reported as one type of plant-growth beneficial bacteria. From the LEfSe analysis, every group owned their biomarkers except VOF. CF and COF owned more biomarkers than other groups, which further verified a significant difference in bacterial community diversity among CF, COF, and the other three groups. Based on the significance analysis for the bacterial community at the phylum and genus level, some beneficial bacteria (e.g., Firmicutes, *Streptomyces, Bacillus*, *Arthrobacter,* and *Paenibacillus*) increased in the four FOF treatments, which was in line with the results of Wu et al. (2014). *Streptomyces* can control tomato diseases by producing antibiotics (Elabyad et al., 1993). Firmicutes, *Bacillus*, and *Paenibacillus* take part both in suppressing plant pathogens and promoting plant growth (Aliye et al., 2008; Algam et al., 2010). Therefore, FOFs supported the disease-suppressing and plant growth-promoting bacteria in the rhizosphere soil, which contributed to tobacco growth.

### 4.4 The relationships between soil bacterial community and environmental factors under different treatments

From the Spearman correlation analysis and RDA between the bacterial community and soil environmental factors, soil TC, TN, CEC, pH, and AP were the most important factors influencing bacterial composition. Simultaneously, the correlation pattern between soil TC and microbiomes was similar to that between soil TN and microbiomes. Soil C, N, and P provide soil microbiomes with principal nutrients and energy sources, which are the common environmental factors that are related to microbial composition (Liu et al., 2018; Liu et al., 2020; Zhao et al., 2020). The demand competition, acquisition, and utilization of C and N are synchronous in microorganisms, resulting in a similar relationship pattern between soil C, N, and microbiomes. There was an apparent improvement in soil AP content, which caused the change of microbiomes to some degree. It is well-known that soil pH is a crucial factor that influences microbial composition and diversity in soil physicochemical properties (Chen et al., 2018b; Bai et al., 2019; Shen et al., 2022). Specifically, soil pH takes a role in bacterial diversity by changing their osmotic pressure and surface potential directly, and indirectly the bioavailability of nutrients and the habitation conditions (Chen W et al., 2018; Bai et al., 2019). Some bacterial genera related to nutrient transition and uptake were enriched in FOF treatments (e.g., genera *Streptomyces* and *Bacillus*). It is reported that *Streptomyces* can stimulate plant growth by fixing atmospheric N into soil NH_4_^+^ and *Bacillus* can improve the soil AK content by effectively dissolving K minerals in soil (Sattar et al., 2019; Liu et al., 2020). Although positive correlations between *Streptomyces* and soil NH_4_^+^, and between *Bacillus* and soil AK were observed in the present study, they did not reach a significant level. From the RDA, tobacco leaf yield showed a positive relationship with soil pH, TC, NH_4_^+^-N, and AP content, and the relative abundances of *Actinobacteriota* and *Proteobacteria*. The increase of soil C, NH_4_^+^-N, and AP content contributed to the accumulation of tobacco leaf biomass, with NH_4_^+^-N extinguishing the most significant improvement effect, which is verified in our study. A significant relationship was found between the rice yield and soil chemical properties, except for AK content (Lv et al., 2011). Wang et al. (2021) also revealed that cucumber yield was significantly positively correlated with soil EC, pH, AP, and DOC content (*p* < 0.01), and soil NH_4_^+^-N content indirectly contributed to cucumber yield with a positive effect on bacterial numbers.

In summary, the mechanisms of FOFs on tobacco yield and quality improvement can be linked to the direct function of the plant growth-promoting substances in FOFs, and the indirect function through altering soil nutrient conditions and bacterial communities. First, FOFs changed the soil nutrient conditions (i.e., increased soil NH_4_^+^-N, AP, AK, TOC, and OM content), which could enable tobacco to absorb more nutrients, and enable the soil to be used more progressively and sustainably. Second, FOFs decreased the accumulation of rhizospheric PA, primarily sinapic acids, which weakened the PA’s allelopathy function. Third, FOFs changed the rhizospheric bacterial community structures, with an increase in beneficial bacteria and a decrease in harmful bacteria, which encouraged the normal growth of tobacco. This study has discussed the effects of different types of FOFs on continuous cropping tobacco yield and quality, focusing on the variance of soil physicochemical properties, the accumulation of PA, and the bacterial community structure. The application of FOFs can be extended to other broad crops at different experimental sites with a larger time scale in a further study. The interactions among FOF applications, the shift of soil environmental factors, soil microbial communities (bacteria, fungi, protists, nematodes), and crop yield, quality, and disease-suppressing ability need to be considered and revealed using various technologies. An antagonistic effect in compound FOF is observed in the present study, and the corresponding mechanisms may emerge from carefully designed experiments.

## 5 Conclusion

This study has shown that the application of different FOFs can improve the agronomic traits, yield, value, and chemical qualities of continuous cropping tobacco when compared to chemical fertilizers in a field experiment. Strikingly, the application of vermicompost-based and wood biochar-based FOFs showed a preferable efficacy. The FOFs took a positive role, primarily by adjusting the soil’s physicochemical properties, decreasing the accumulation of PA (especially sinapic acid), and inducing a healthier rhizospheric bacterial community structure. Additionally, soil AP, TC, TN, CEC, and pH were the important environmental factors that influenced the bacterial community structure. Tobacco yield showed an active correlation with soil TC, NH_4_^+^-N, and AP content, and with the relative abundances of *Proteobacteria* and *Actinobacteriota*. This study provides insights into enhancing tobacco yield and chemical quality by applying proper FOFs as basal fertilizers to improve the soil’s micro-ecology environment.

## Data Availability

The original contributions presented in the study are publicly available. The raw sequence data were deposited in the NCBI Sequence Read Archive (SRA) database: https://www.ncbi.nlm.nih.gov/srawith. Accession number PRJNA837398.
