# Supplementary material for: Functional organic fertilizers can alleviate tobacco (Nicotiana tabacum L.) continuous cropping obstacle via ameliorating soil physicochemical properties and bacterial community structure
Source: Front Bioeng Biotechnol. 2022 Oct 20;10:1023693. doi: 10.3389/fbioe.2022.1023693 (PMC9631321; doi:10.3389/fbioe.2022.1023693)
Supplement: Supplementary file 1 [file DataSheet1.docx]

# Functional organic fertilizers can alleviate tobacco (*Nicotiana tabacum* L.) continuous cropping obstacle via ameliorating soil physicochemical properties and bacterial community structure

**Dan Chen^1^, Mei Wang^1^, Gang Wang^1^, Yujie Zhou^1^, Jiangzhou Li^2^, Cuiping Zhang^2^, Xiaoe Yang^1*^, Kuai Dai^2*^**

1Ministry of Education (MOE) Key Laboratory of Environment Remediation and Ecological Health, College of Environmental and Resource Sciences, Zhejiang University, Hangzhou 310058, People’s Republic of China

2Yuxi Tobacco Company, Ltd. of Yunnan Province, Yuxi 653100, People’s Republic of China

**^*^ Correspondence:**

Xiaoe Yang: [xeyang@zju.edu.cn](mailto:xeyang@zju.edu.cn); Kuai Dai: [daikuai520@163.com](mailto:daikuai520@163.com)

**Supplementary Figures**

**
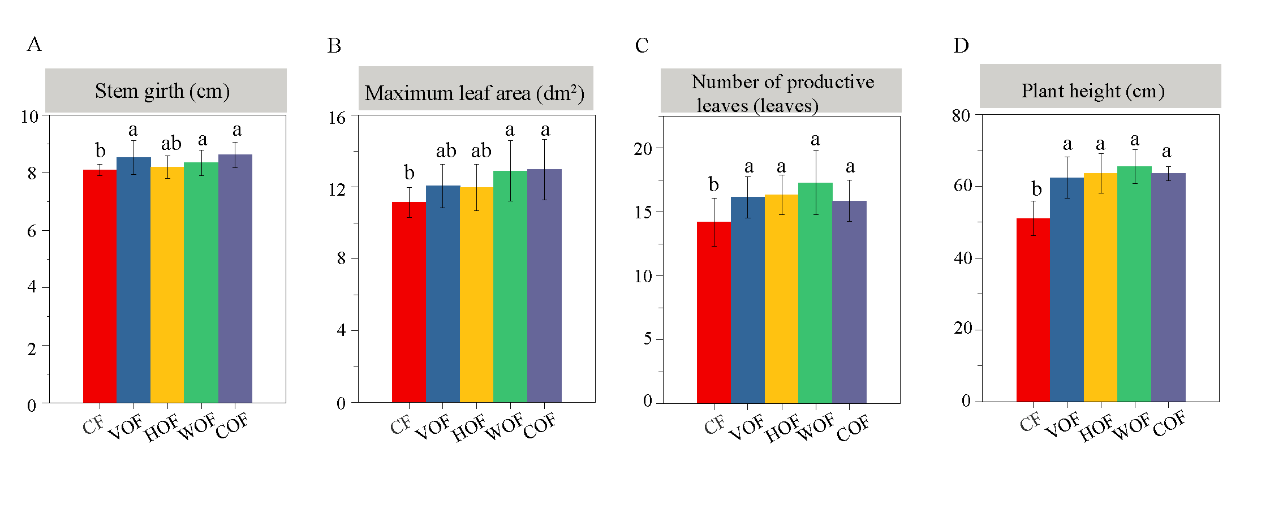
Fig. S1.** Tobacco agronomic characteristics. CF, VOF, HOF, WOF, and COF represent the treatments of tobacco-special chemical fertilizer, vermicompost-based, humic acid-based, wood biochar-based and compound functional organic fertilizers, respectively. The maximum leaf area = 0.6345 × largest leaf width × largest leaf length. Different letters within each index show a significant difference based on the Duncan’s method (p < 0.05).

**
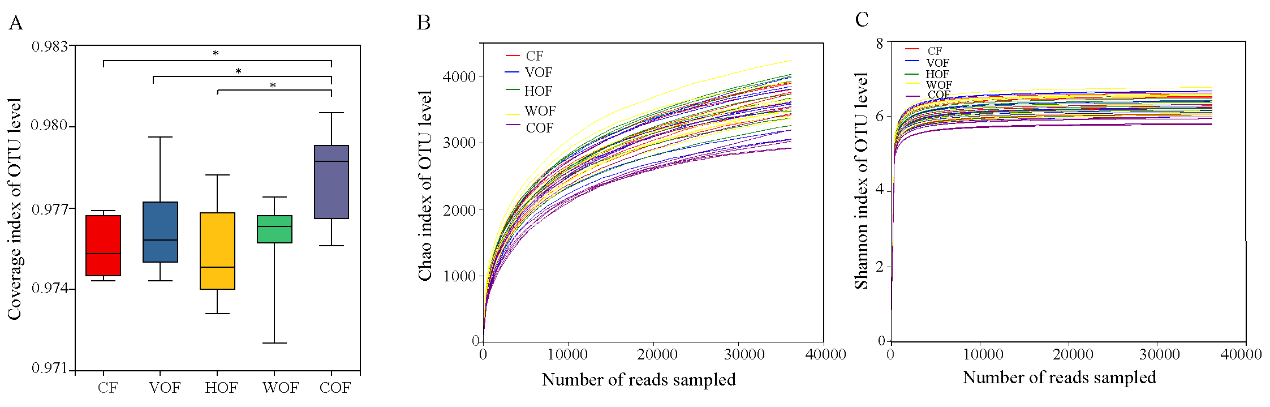
**

**Fig. S2.** The Coverage index (**A**), the rarefaction curve of Chao index (**B**) and Shannon index (**C**) of OTU level. CF, VOF, HOF, WOF, and COF represent the treatments of tobacco-special chemical fertilizer, vermicompost-based, humic acid-based, wood biochar-based and compound functional organic fertilizers, respectively.


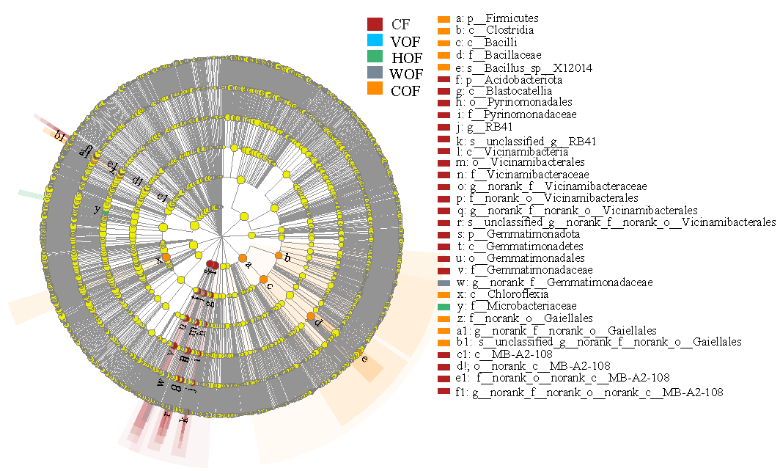


**Fig. S3.** LEfSe cladogram showing bacterial taxa with different abundance values (LDA score > 3.5, *p* < 0.05) in the rhizosphere soil. CF, VOF, HOF, WOF, and COF represent the treatments of tobacco-special chemical fertilizer, vermicompost-based, humic acid-based, wood biochar-based and compound functional organic fertilizers, respectively.


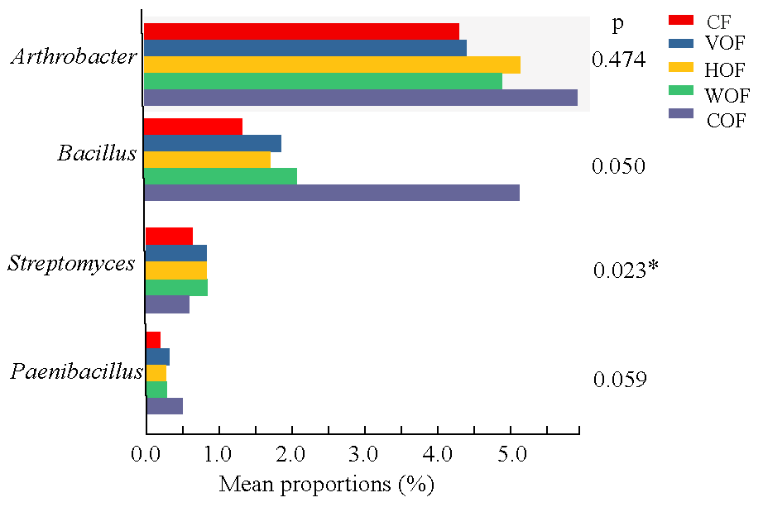


**Fig. S4.** The comparative analysis of some bacterial community at the genus level in the rhizosphere soil based on the Kruskal-Wallis H test. CF, VOF, HOF, WOF, and COF represent the treatments of tobacco-special chemical fertilizer, vermicompost-based, humic acid-based, wood biochar-based and compound functional organic fertilizers, respectively.
